# A Specific for Singultus

**Published:** 1884-02

**Authors:** 


### A Specific for Singultus.

Says Dr. Henry Tucker, in *South Medical Record*: This very common affection, of infants and children especially, has a specific remedy, at least one which I have never known to fail. Moisten granulated sugar with good cider vinegar; give to an infant from a few grains to a teaspoonful. The effect is almost instantaneous, and the dose seldom needs to be repeated. I have used it for all ages, from infants a few months old to those on the down-hill side of life.

